# Developing a simple method to enhance the generation of cone and rod photoreceptors in pluripotent stem cell‐derived retinal organoids

**DOI:** 10.1002/stem.3082

**Published:** 2019-10-31

**Authors:** Darin Zerti, Birthe Dorgau, Majed Felemban, Ali E. Ghareeb, Min Yu, Yuchun Ding, Natalio Krasnogor, Majlinda Lako

**Affiliations:** ^1^ Institute of Genetic Medicine Newcastle University Newcastle upon Tyne UK; ^2^ Princess Al‐Jawhara Center of Excellence in Research of Hereditary Disorders and Department of Medical Technology, Faculty of Applied Medical Sciences King Abdulaziz University Jeddah Saudi Arabia; ^3^ Interdisciplinary Computing and Complex Biosystems (ICOS) Research Group Newcastle University Newcastle upon Tyne UK

**Keywords:** retinal organoids, photoreceptors, pluripotent stem cells

## Abstract

Cell replacement therapy is a promising treatment for irreversible retinal cell death in diverse diseases such as Stargardt's disease, age‐related macular degeneration, and retinitis pigmentosa. The final impact of all retinal dystrophies is the loss of photoreceptors; hence, there is a pressing need for research into replacement. Seminal work has shown that a simple three‐dimensional culture system enables differentiation of human pluripotent stem cells to retinal organoids containing large numbers of photoreceptors developing alongside retinal neurons and Müller glia cells in a laminated structure that resembles the native retina. Despite these promising developments, current protocols show different efficiencies across pluripotent stem cells and result in retinal organoids with a mixture of photoreceptor cells at varying maturation states, along with nonphotoreceptor cell types. In this study, we investigated the impact of stage‐specific addition of retinoic acid (RA), 9‐cis‐retinal, 11‐cis‐retinal, levodopa (l‐DOPA), triiodothyronine (T3), and γ‐secretase inhibitor ((2S)‐N‐[(3,5‐Difluorophenyl)acetyl]‐l‐alanyl‐2‐phenyl]glycine1,1‐dimethylethyl ester2L [DAPT]) in the generation of cone and rod photoreceptors. Our results indicate that addition of RA + T3 during days 90 to 120 of differentiation enhanced the generation of rod and S‐cone photoreceptor formation, while the combined addition of DAPT from days 28 to 42 with RA during days 30 to 120 of differentiation led to enhanced generation of L/M‐cones at the expense of rods. l‐DOPA when added together with RA during days 90 to 120 of differentiation also promoted the emergence of S‐cones at the expense of rod photoreceptors. Collectively, these data represent an advance in our ability to direct generation of rod and cone photoreceptors in vitro.

## Graphical abstract

Schematic summary of our results showing that the addition of retinoic acid + T3 (days 90‐120) led to enhanced generation of rod and S‐cone photoreceptors while combined addition of retinoic acid + Levodopa, at the same time window, promoted only the S‐cone formation. The supplementation of retinoic acid + DAPT (days 30‐120 for RA+ days 28‐42 for DAPT) resulted in an increased L/M‐cone photoreceptor generation.

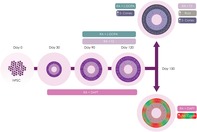

**Significance statement**

The generation of retinal organoids from human pluripotent stem cells provides an in vitro model for disease modeling and replacement therapies. To date, the efficiency of protocols for generating retinal organoids is variable, resulting in the emergence of all retinal cell types, including photoreceptors, but in different ratios and at different maturation stages. Our data show that the addition of retinoic acid in combination with T3, levodopa, or (2S)‐N‐[(3,5‐Difluorophenyl)acetyl]‐L‐alanyl‐2‐phenyl]glycine1,1‐dimethylethyl ester2L at specific time intervals promotes cone and/or rod formation.

## 1 INTRODUCTION

Degenerative diseases of the retina represent one of the main causes of visual impairment and blindness, which culminate in the loss of photoreceptors and remain incurable.^1^ Cell transplantation approaches for the replacement of lost or damaged photoreceptors have been investigated over the last decades in preclinical animal models of retinal degeneration with some promising results^2^; suggesting that cell replacement therapies may be feasible for the treatment of retinal dystrophies. Human embryonic stem cells (hESCs)^3^ and induced pluripotent stem cells (hiPSCs)^4^ provide a suitable tool because both cell types can be expanded indefinitely and have the capacity to produce cone and rod precursors as well as more mature photoreceptor progeny in vitro.^5^ The ability to generate retinal organoids from hESCs/hiPSCs under three‐dimensional culture conditions has been a great advance toward the generation of clinically relevant cell populations that closely follow in vivo retinogenesis.^6^, ^7^ In the last 7 years, intense work has been performed by several groups worldwide to improve the robustness and efficiency of differentiation protocols and to understand the factors and signaling pathways that are required to enhance retinal specification.^8^, ^9^ As such, developing methods to modulate cell composition and align differentiation states in vitro is critical for the path toward clinical transplantations. In this study, we investigated the impact of stage‐specific addition of six reagents and established a simple method to enhance the generation of cone or rod photoreceptors in vitro.

## 2 MATERIALS AND METHODS

A detailed description of all experimental procedures is presented in the online Supporting Information.

## 3 RESULTS AND DISCUSSION

**Figure 1:**
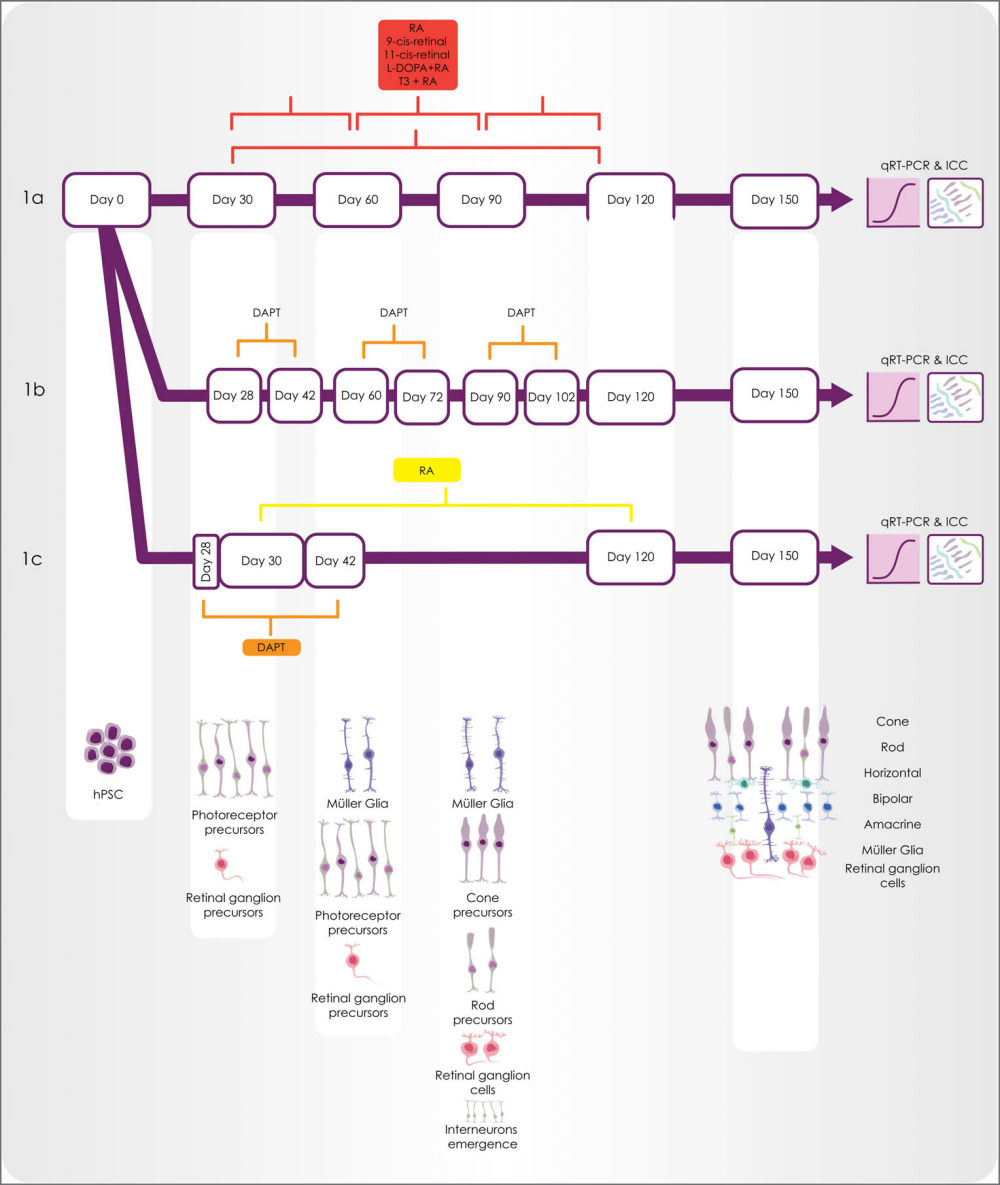
Diagram of the experimental design, showing the addition of retinoic acid, 9‐cis‐retinal, 11‐cis‐retinal, levodopa, triiodothyronine, and DAPT individually or in combination with each other as well as the emergence of retinal cell types at different time points during the differentiation of retinal organoids

**Figure 2:**
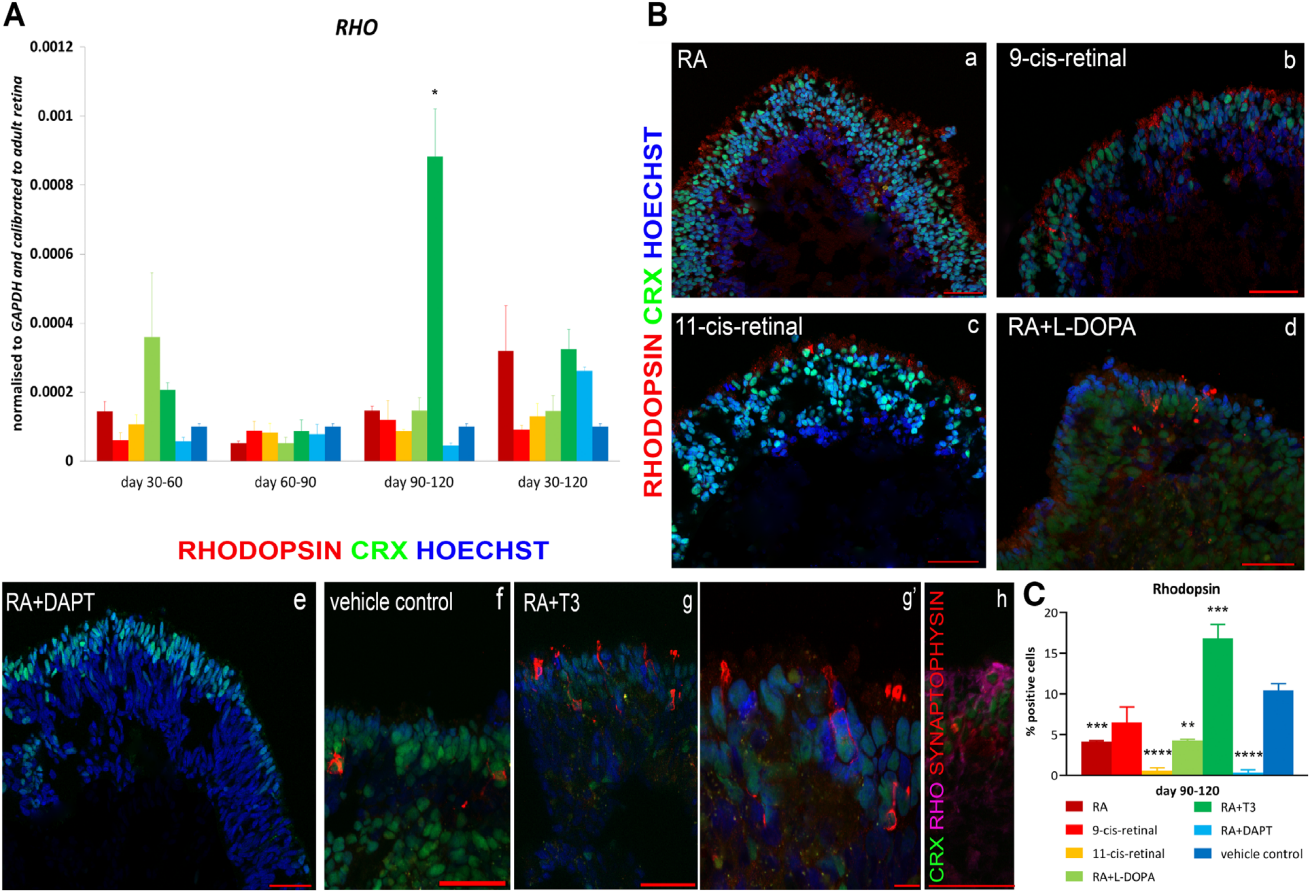
Characterization of Rhodopsin expression in retinal organoids derived from human embryonic stem cells at day 150 of differentiation. A, Gene expression analysis of *Rhodopsin* at all time points during differentiation revealed a significant increase in RA + T3 days 90 to 120 condition compared with vehicle control at days 90 to 120. B, Representative examples of Rhodopsin immunoreactivity (red) in all conditions (a–g) from days 90 to 120 stage‐specific additions, showing the highest number of Rhodopsin^+^ cells in RA + T3 condition (g). Higher magnification demonstrated the typical morphology of rod photoreceptors (g′). Double staining with Rhodopsin (magenta) and Synaptophysin (red) indicated the possible formation of synapses in the developing OPL (h). CRX (green) represents the endogenous GFP expression and nuclei are counterstained with Hoechst (blue). Scale bars: 50 μm (Ba–h), 10 μm (Bg′). C, Immunohistochemistry quantification revealed a reduction of Rhodopsin^+^ cells in RA, 11‐cis‐retinal, RA + l‐DOPA, and RA + DAPT condition and a significant increase in RA + T3 condition compared with vehicle control. Data are shown as mean ± SEM (*n* = 5) and statistical significant differences were considered at *P < .05, **P < .01, ***P < .001, ****P < .0001. Abbreviations: CRX, cone rod homeobox; GFP, green fluorescent protein; l‐DOPA, levodopa; OPL, outer plexiform layer; RA, retinoic acid; RHO, Rhodopsin; T3, triiodothyronine

The CRX‐GFP (H9) hESC line was expanded and differentiated to retinal organoids, which were collected at day 150 and processed for quantitative reverse transcription polymerase chain reaction (qRT‐PCR) and immunohistochemistry. The following reagents: retinoic acid (RA), 9‐cis‐retinal, 11‐cis‐retinal, levodopa (l‐DOPA), triiodothyronine (T3), and (2S)‐N‐[(3,5‐Difluorophenyl)acetyl]‐l‐alanyl‐2‐phenyl]glycine1,1‐dimethylethyl ester2L (DAPT) were added at specific time intervals during differentiation as shown in Figure 1. All these supplements have been shown to promote cone or rod photoreceptor formation in other species. For example, RA enhances rod differentiation in vivo and in vitro,^10^, ^11^ expression of rod photoreceptor transcription factor neural retina leucine zipper,^12^ and red cone opsin development in vivo.^13^ T3 is also required for rod photoreceptor development in vivo,^14^ regulating the ratio and patterning of cone opsin expression,^15^ specifying cone subtype generation^16^ as well as suppressing cone viability.^17^ In accordance with these published data, the combined addition of RA and T3 during days 90 to 120 of differentiation resulted in the highest gene expression of *Rhodopsin* (rod marker; Figure 2A). These results were further corroborated by the immunohistochemical analysis, which revealed a significantly higher number of rods (identified by the marker protein Rhodopsin), compared with the control group (vehicle alone) and the groups supplemented with 9‐ or 11‐cis retinal, RA, RA + l‐DOPA, or RA + DAPT (Figure 2Ba–f,2C and Figure S1). 9‐cis‐retinal has been shown to encourage rod differentiation in developing mammalian retina.^18^, ^19^ 11‐cis‐retinal, the light‐sensitive component of rod and cone photoreceptors, has been shown to activate Rhodopsin expression in vitro.^20^ Despite these findings, our data show that the number of Rhodopsin positive cells were lower in RA alone, 9‐ and 11‐cis retinal (Figure 2Ba–f,C) when compared with the control group. In all conditions, rods were located in the apical layer of the retinal organoids, forming a putative outer nuclear layer (ONL): furthermore, higher magnification observations indicated the typical morphology of mature rods (Figure 2Bg–g′). Synaptic connections between mature rod photoreceptors and second order neurons (horizontal and bipolar cells) within the outer plexiform layer (OPL) were observed by double staining with Rhodopsin and Synaptophysin, a marker for synapses, showing Synaptophysin expression underneath the ONL of retinal organoids (Figure 2Bh and Figure S1).

**Figure 3:**
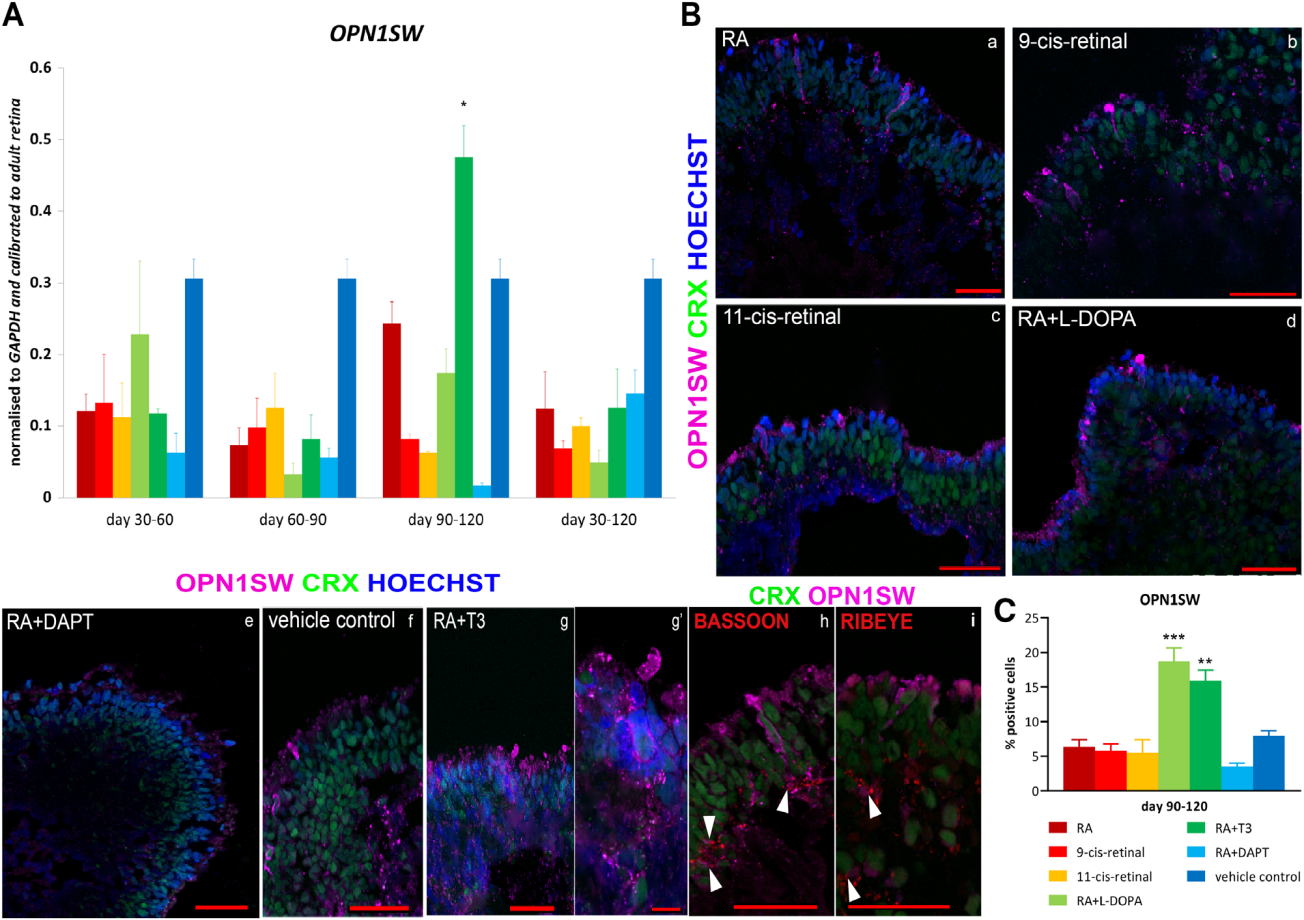
Characterization of OPN1SW (S cones) expression in retinal organoids derived from human embryonic stem cells at day 150 of differentiation. A, qRT‐PCR analysis of *OPN1SW* at all time points during differentiation showed an increase in RA + T3 condition compared with vehicle control (*P* < .05) at days 90 to 120. B, Expression of OPN1SW (magenta) was found in all conditions at time point days 90 to 120 (a–g). The highest number of OPN1SW^+^ cells was observed in RA + T3 and RA + l‐DOPA conditions (g, d) highlighting cells in the higher magnification image (g′). Double staining with OPN1SW (magenta) and Bassoon (red; h) or Ribeye (red; i), respectively, indicated putative ribbon synapse formation in the developing OPL (arrowheads). CRX (green) represents the endogenous GFP expression and nuclei are counterstained with Hoechst (blue). Scale bars: 50 μm (Ba–i), 10 μm (Bg′). C, Immunohistochemistry quantification analysis showed a significant increase of OPN1SW^+^ cells in RA + l‐DOPA and RA + T3 condition compared with vehicle control. Data are shown as mean ± SEM (*n* = 5) and statistical significant differences were considered at *P < .05, **P < .01, ***P < .001. Abbreviations: CRX, cone rod homeobox; GFP, green fluorescent protein; l‐DOPA, levodopa; OPL, outer plexiform layer; RA, retinoic acid; T3, triiodothyronine

Addition of RA and T3 also resulted in the highest expression of S‐cone photoreceptor marker (*OPN1SW*; Figure 3A). Immunohistochemical analysis confirmed these findings, but also highlighted another group (RA + l‐DOPA) to be equally efficient for the generation of S cones (Figure 3B,C, Figures S2 and S3). l‐DOPA is produced by the retinal pigment epithelium: its absence results in reduced rod numbers with no effect on the cone population in mouse ESC (mESC).^21^ In contrast, our results in the human system revealed that the addition of RA + l‐DOPA during days 90 to 120 of differentiation led to the enhancement of S‐cone formation at the expense of rods (Figure 3C and Figure S3). In this group, possible synapse formation between OPN1SW^+^ cells and second order neurons within the putative developing OPL was confirmed by the expression of Bassoon, an essential component of the ribbon synapses (Figure 3Bh, arrowheads) and Ribeye, the main protein component of synaptic ribbons (Figure 3Bi arrowheads).

**Figure 4:**
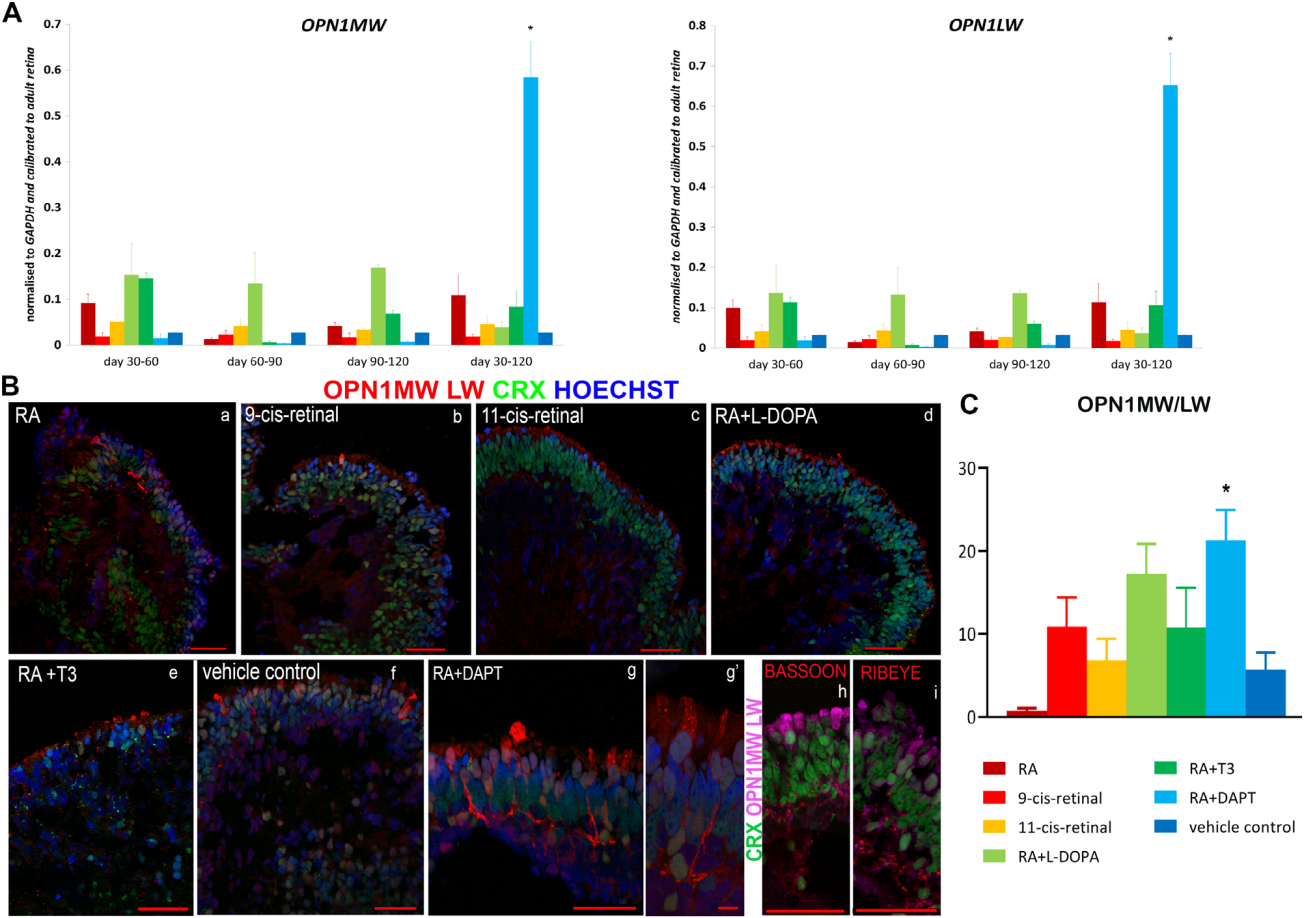
Characterization of OPN1MW/LW (L/M cones) expression in retinal organoids derived from human embryonic stem cells at day 150 of differentiation. A, Gene expression analysis of *OPN1MW* (left) and OPN1LW (right) at all time points during differentiation revealed a significant increase in the expression of both genes in RA + DAPT condition compared with vehicle control at days 30 to 120. B, Representative examples of OPN1MW/LW expression (red) in all conditions at time point days 30 to 120 (a–g), indicating the highest number OPN1MW/LW^+^ cells in RA + DAPT condition (g). Higher magnification demonstrated the morphology of L/M cones g′, Ribbon synapse formation was demonstrated by double staining of OPN1MW/LW (magenta) with Bassoon (red; h) or Ribeye (red; i), respectively. CRX (green) represents the endogenous GFP expression and nuclei are counterstained with Hoechst (blue). Scale bars: 50 μm (Ba–i), 10 μm (Bg′). C, Quantification of OPN1MW/LW^+^ cells revealed a significant increase of L/M cones in RA + DAPT condition compared with vehicle control. Data are shown as mean ± SEM (*n* = 5) and statistical significant differences were considered at *P < .05. Abbreviations: CRX, cone rod homeobox; GFP, green fluorescent protein; l‐DOPA, levodopa; RA, retinoic acid; T3, triiodothyronine

The γ‐secretase inhibitor DAPT, a known Notch signaling inhibitor, has been reported to increase cone or rod cell differentiation when added at specific stages of mESC differentiation.^22^, ^23^ Our qRT‐PCR analysis showed the highest expression of *OPN1MW* (M‐cone photoreceptor marker) and *OPN1LW* (L cone photoreceptor marker) in the retinal organoids that had been exposed to the combined addition of RA + DAPT from days 30 to 120 of differentiation (Figure 4A). Additionally, immunohistochemical analysis and its quantification indicated a significant increase in the percentage of mature L/M cones in this group at the expense of rods compared with the vehicle control (Figure 4Ba–g′,C and Figures S3 and S4). In comparison to the vehicle control alone, all culture conditions revealed a slightly higher expression of OPN1MW/LW^+^ cells, except for the RA condition (Figure 4C). Furthermore, the formation of cone pedicles in the putative OPL, where they form ribbon synapses with Horizontal/Bipolar cells, was demonstrated by the immunoreactivity of Bassoon and Ribeye, respectively (Figure 4Bh–i). Interestingly, in contrast to Nakano et al.,^6^ the DAPT treatment did not interfere with the tissue architecture and the lamination of the retinal organoids (Figures S5 and S6). More extensive comparisons with work performed by others on the impacts of DAPT on retinal cell formation and maturation are not possible, as different groups use different media compositions for the culture of retinal organoids. Nonetheless, our study shows an advance in the field, as we were able to demonstrate that addition of RA and T3 results in generation of all cone subtypes as well as rods at day 150 of differentiation, unlike the Eldred et al. study where by day 150 of differentiation only S cones were found within the retinal organoids with L/M cones and rods observed later during the differentiation process.^16^

## 4 CONCLUSION

The results of this study indicate that addition of RA + T3 at specific stages of differentiation of retinal organoids, leads to enhanced generation of rod and S‐cone photoreceptors, which were able to form synaptic connections with the appropriate interneurons in the putative OPL. Combined additions of RA + l‐DOPA or RA + DAPT led to selective enhancement either of S‐cone photoreceptor or L/M‐cone photoreceptor generation, respectively, at the expense of rod formation. Together, our data show that addition of specific reagents at selected differentiation time points can provide a useful strategy for the generation of retinal organoids enriched for specific photoreceptor subtype of interest.

## AUTHOR CONTRIBUTIONS

D.Z.: performed research, data collection and analysis, figure preparation and manuscript writing, approved the final version of the manuscript. B.D.: data analysis, figure preparation and contributed to manuscript writing, approved the final version of the manuscript. M.F., A.E.G.: performed research and data collection, approved the final version of the manuscript. M.Y.: performed research, approved the final version of the manuscript. Y.D., K.N.: data analysis, approved the final version of the manuscript. M.L.: study design, data analysis, figure preparation, manuscript writing and fund raising, approved the final version of the manuscript.

## CONFLICT OF INTEREST

The authors indicated no potential conflicts of interest.

## Supporting information

Supporting information.

**Supplementary Table S1** Definition of Basal Media during the differentiation

**Supplementary Table S2** List of Primary Antibodies used for the IHC

**Supplementary Table S3** List of primers used for the qRT‐PCR

**Figure S1** Individual channels images of Rhodopsin immunoreactivity (red), CRX (green) represents the endogenous GFP expression and nuclei are counterstained with Hoechst (blue) in all conditions (A‐H) from day 90‐120 stage‐specific additions, showing the highest number of Rhodopsin^+^ cells in RA + T3 condition (F, F′ and F″). Higher magnification showed the nuclei in blue (G), the endogenous GFP expression in the apical layer (G’) and the typical morphology of rod photoreceptors (G”). Individual channel of the double staining with Rhodopsin (magenta) and Synaptophysin (red) indicated the possible formation of synapses in the developing OPL (I and I″); CRX (green) represents the endogenous GFP expression (I′). Scale bars 50 μm (A‐I″).

**Figure S2** Individual channels images of OPN1SW immunoreactivity (magenta), CRX (green) represents the endogenous GFP expression and nuclei are counterstained with Hoechst (blue) in all conditions (A‐H) from day 90‐120 stage‐specific additions, showing the highest number of OPN1SW^+^ cells in RA + T3 condition (G, G’ and G”). Higher magnification showed the nuclei in blue (H), the endogenous GFP expression in the apical layer (H′) and the typical morphology of S‐cone photoreceptors (H″). Individual channels of the double staining with OPN1SW (magenta) and Bassoon (red) (I and I″); OPN1SW (magenta) and Ribeye (red) (J and J”) indicated, respectively the possible formation of synapses in the developing OPL; CRX (green) represents the endogenous GFP expression (I′ and J’). Scale bars 50 μm (A, A’, A”, B, B′, B″, C, C′, C″, D, D’, D”, E, E’, E”, F, F′, F″, G, G’, G”, I, I′, I″, J, J’ and J”) and 10 μm (H, H′ and H″).

**Figure S3** Diagram showing the percentage of photoreceptors within the retinal organoids divided into S cones (blue), L/M cones (green) and rods (red) in all conditions. Data are presented as average of two time points: day 90‐120 and day 30‐120.

**Figure S4** Individual channels images of OPN1MW/LW immunoreactivity (red), CRX (green) represents the endogenous GFP expression and nuclei are counterstained with Hoechst (blue) in all conditions (A‐H) from day 30‐120 stage‐specific additions, showing the highest number of OPN1MW/LW^+^ cells in RA + DAPT condition (G, G’ and G”). Higher magnification showed the nuclei in blue (H), the endogenous GFP expression in the apical layer (H′) and the typical morphology of L/M‐cone photoreceptors (H″). Individual channels of the double staining with OPN1MW/LW (magenta) and Bassoon (red) (I and I″); OPN1MW/LW (magenta) and Ribeye (red) (J and J”) indicated the possible formation of synapses in the developing OPL. CRX (green) represents the endogenous GFP expression (I′ and J’). Scale bars 50 μm (A, A’, A”, B, B′, B″, C, C′, C″, D, D’, D”, E, E’, E”, F, F′, F″, G, G’, G”, I, I′, I″, J, J’ and J”) and 10 μm (H, H′ and H″).

**Figure S5** Characterization of retinal organoid lamination in RA + DAPT condition at day 150 of differentiation. **A**) Representative brightfield images of retinal organoids at day 150, showing bright phase neuroepithelium on the apical side of organoids. **B**) CRX (endogenous GFP expression; green) and Recoverin (red) expression was found at the apical edge of organoids, forming a putative ONL. **C**) Some NRL^+^ cells (red) were found in the photoreceptor layer at the apical edge of retinal organoids. **D**) Expression of Arrestin3 (red) was observed above photoreceptor nuclei in the developing photoreceptor inner/outer segments. **E**) RXRγ^+^ cells (red) were seen throughout the retinal organoid. **F**) Ganglion cells detected by HuC/D (red) were located in the middle of retinal organoids, forming a putative GCL. CRX (green) represents the endogenous GFP expression and nuclei are counterstained with Hoechst (blue). Abbreviations: ONL, outer nuclear layer; GCL, ganglion cell layer. Scale bars, 200 pixel (A) and 50 μm (B‐F). Abbreviations: RA, Retinoic Acid.

**Figure S6** Brightfield representative examples of retinal organoids at day 150 of differentiation for RA + T3 (**A‐D**) and RA + DAPT (**E‐H**) conditions. In both conditions, neural retinal and RPE are visible. Scale bar, 200 pixel. Abbreviations: RA, Retinoic Acid; T3, triiodothyronine.
