# Supplementary material for: Developing a simple method to enhance the generation of cone and rod photoreceptors in pluripotent stem cell‐derived retinal organoids
Source: Stem Cells. 2019 Oct 31;38(1):45–51. doi: 10.1002/stem.3082 (PMC7004057; doi:10.1002/stem.3082)
Supplement: Supplementary file 1 — Supporting information. [file STEM-38-45-s001.docx]

## Human Pluripotent Stem Cells differentiation to retinal organoids

Retinal organoids were differentiated from the CRX-GFP H9 human ESC cell line using a feeder-free system [1–2]. ESCs were expanded in mTeSR™1 (StemCell Technologies, 05850) at 37°C and 5% CO_2_ on 6 well plates pre-coated with Low Growth Factor Matrigel (Corning, 354230). The retinal organoids were generated following a protocol described in Collin et al., 2019 [2] with addition of various supplements, which are shown in **Figure 1**. The composition of the basal media for each stage of differentiation is shown in the **Table S1**. The added supplements include Retinoic Acid (RA) (0.5 μM, Sigma-Aldrich UK), 9-cis-retinal (0.5 μM, Sigma-Aldrich UK), 11-cis-retinal (0.5 μM, BOC Sciences), Levodopa (0.5 μM, Sigma-Aldrich UK), Triiodothyronine (T3) (40 ng/ml, Sigma-Aldrich) and DAPT (10 μM Sigma-Aldrich UK) singly or in combination with each other from day 18 of differentiation for specific durations (day 30-60, day 60-90, day 90-120 and day 30-120), except for DAPT, which was applied both alone and in combination with RA (**Figure 1**).

## Immunohistochemistry

Retinal organoids were collected on day 150 and fixed in 2% PFA for 30 minutes, followed by three washes in Phosphate-buffered saline (PBS), incubated overnight in 30% sucrose in PBS, embedded in Optical Cutting medium (OCT) (Cellpath, UK) and frozen at -20°C. Ten-micrometre cryostat sections were collected using a Leica Cm1860 cryostat (Leica, Germany) onto Superfrost Plus slides and stored at -20°C in slide boxes prior to immunostaining. Cryosections were air-dried, washed several times in PBS and incubated in blocking solution (10 % normal goat serum, 0.3 % Triton-X-100 in PBS) for one hour at room temperature. Slides were incubated with the appropriate primary antibody overnight at 4°C (**Table S2**). After rinsing with PBS, sections were incubated with the secondary antibody for 2 hours at room temperature. Alexa Fluor 647 and 546 secondary antibodies (Invitrogen-Molecular Probes) were used at a 1:1000 dilution. Negative controls were carried out by omitting the primary antibody. Afterwards, sections were washed three times in PBS and mounted with Vectashield (Vector Laboratories, Burlingame, CA) containing Hoechst (Life Technologies, UK).

## Image Acquisition and analysis

Images were captured using an Axio Imager upright microscope with Apotome (Zeiss, Germany) structured illumination fluorescence using 20x objective and 63x oil objective operated with AxioVision software. Final images are presented as a maximum projection and adjusted for brightness and contrast in Adobe Photoshop (Adobe Systems).

**Image Quantification**

Cell image quantitation was performed using the MATLAB software (Mathworks, MA) following the protocol described in Dorgau *et al., 2019* [3]. A minimum of eight individual retinal organoids were analysed for each condition and for each biological replicate. Three biological replicates were included. All results were further analysed using Microsoft Excel and Prism (GraphPad, USA).

**qRT-PCR**

Retinal organoids were collected on day 150 and assessed by quantitative RT-PCR; 15-20 retinal organoids for each condition and for each biological replicate were homogenised using a Dounce Tissue Grinder (Sigma-Aldrich, UK) to extract the RNA using the Promega tissue extraction kit (Promega, USA) as per the manufactures instructions. Three biological replicates were included. 1μg of RNA was reverse transcribed using random primers (Promega, USA). qRT-PCR was performed using a Quant Studio 7 Flex system (Applied Biosystems, USA) with SYBR Green reaction mixture (Promega, USA). Each primer (**Table S3**) was used at a concentration of 0.5 µM. The reaction parameters were as follows: 95°C for 15 minutes to denature the cDNA and primers, 40 cycles of 94°C for 15 seconds followed by primer specific annealing temperature for 30 seconds (60°C), succeeded by a melt curve. A comparative cycle threshold (Ct) method was used to calculate the levels of relative expression, whereby the Ct was normalised to the endogenous control (*GAPDH*). This calculation gives the Δ^Ct^ value, which was then normalised to a reference sample (i.e. control group), giving the ΔΔ^Ct^. The fold change was calculated using the following formula: 2-ΔΔ^Ct^.

**Statistical Analyses**

All statistical tests were performed using Prism (GraphPad, USA). The standard errors of all means (SEM) were calculated. Statistical significance was tested using either one-way ANOVA or two-way ANOVA (Dunnett statistical hypothesis for multiple test correction). Asterisk = p-value < 0.05, two asterisks = p-value < 0.01, three asterisks = p-value <0.001, four asterisks = p-value <0.0001.

**References**

1 Collin J, Mellough CB, Dorgau B, et al. Using Zinc Finger Nuclease Technology to Generate CRX-Reporter Human Embryonic Stem Cells as a Tool to Identify and Study the Emergence of Photoreceptors Precursors During Pluripotent Stem Cell Differentiation. Stem Cells 2016;34:311–321.

2 Collin J, Queen R, Zerti D, et al. Deconstructing Retinal Organoids: Single Cell RNA-Seq Reveals the Cellular Components of Human Pluripotent Stem Cell-Derived Retina. Stem Cells 2019.

3 Dorgau B, Felemban M, Hilgen G, et al. Decellularised extracellular matrix-derived peptides from neural retina and retinal pigment epithelium enhance the expression of synaptic markers and light responsiveness of human pluripotent stem cell derived retinal organoids. Biomaterials 2019;199:63–75.
