# Supplementary material for: Developing a simple method to enhance the generation of cone and rod photoreceptors in pluripotent stem cell‐derived retinal organoids
Source: Stem Cells. 2019 Oct 31;38(1):45–51. doi: 10.1002/stem.3082 (PMC7004057; doi:10.1002/stem.3082)
Supplement: Supplementary file 2 — Supplementary Table S1 Definition of Basal Media during the differentiation [file STEM-38-45-s002.docx]

**Supplementary Table 1: Definition of Basal Media during the differentiation**

| **Product** | **Initial**  **Concentration** | **Final Concentration** | **Day 0-1** | **Day 2-17** | **day 18-29** | **day 30- 150** |
| --- | --- | --- | --- | --- | --- | --- |
| mTeSR1 |  |  | v |  |  |  |
| Rock Inhibitor | 10 mM | 10μM | v |  |  |  |
| DMEM/F12-Glutamax |  |  |  | v | v | v |
| FBS |  | 10% |  |  | v | v |
| KnockOut^TM^ Serum Replacement |  | 10% |  | v |  |  |
| Penicillin/Streptomycin | 100x | 1X |  | v | v | v |
| Fungizone | 250 μg/ml | 25 μg/ml |  |  | v | v |
| B27 | 50x | 1X |  | v | v | v |
| IGF-1 | 100 μg/ml | 5 ng/ml |  | v | v |  |
| IGF-1 | 100 μg/ml | 10 ng/ml |  |  |  | v |
| NEEA | 100x | 1X |  | v | v | v |
| N2 | 100x | 1X |  |  |  | v |
| Taurine | 0.1 M | 0.1 mM |  |  | v | v |
| Lipids | 100x | 1X |  |  |  | v |
