# Supplementary material for: Developing a simple method to enhance the generation of cone and rod photoreceptors in pluripotent stem cell‐derived retinal organoids
Source: Stem Cells. 2019 Oct 31;38(1):45–51. doi: 10.1002/stem.3082 (PMC7004057; doi:10.1002/stem.3082)
Supplement: Supplementary file 3 — Supplementary Table S2 List of Primary Antibodies used for the IHC [file STEM-38-45-s003.docx]

| **Antibody** | **Host** | **Dilution** | **Supplier, Cat. No** |
| --- | --- | --- | --- |
| Anti-opsin Blue (OPN1SW) | Rabbit | 1:200 | Millipore, AB5407 |
| Anti-opsin Red/Green (OPN1LW/MW) | Rabbit | 1:200 | Millipore, AB5405 |
| Anti-RetP1(Rhodopsin) | Mouse | 1:200 | Sigma, O4886 |
| Anti-Bassoon | Mouse | 1:100 | StressGen, VAM-PS003 |
| Anti-Ribeye | Mouse | 1:100 | BD Bioscience, 612044 |
| Anti- Synaptophysin | Rabbit | 1:200 | abcam, ab32127 |

**Supplementary Table 2: List of Primary Antibodies used for the IHC**
