# Supplementary material for: Developing a simple method to enhance the generation of cone and rod photoreceptors in pluripotent stem cell‐derived retinal organoids
Source: Stem Cells. 2019 Oct 31;38(1):45–51. doi: 10.1002/stem.3082 (PMC7004057; doi:10.1002/stem.3082)
Supplement: Supplementary file 4 — Supplementary Table S3 List of primers used for the qRT‐PCR [file STEM-38-45-s004.docx]

**Supplementary Table 3: List of primers used for the qRT-PCR**

| Gene | **Forward Primer**  (Sequence (5'->3') | **Reverse Primer**  (Sequence (5'->3') | **NCBI Reference Sequence** |
| --- | --- | --- | --- |
| *GAPDH* | TGCACCACCAACTGCTTAGC | GGCATGGACTGTGGTCATGAG | NM_001256799.2 |
| *OPSINLW* | GCCTACTTTGCCAAAAGTGC | GATGAGACCTCCGTTTTGGA | NM_020061 |
| *OPSINMW* | CATCTTTGGTTGGAGCAGGTACT | TCTCTGCCTTCTGGGTGGAT | NM_000513.2 |
| *OPSINSW* | ATACCGCAGCGAGTCCTATAC | GATCCTACCATCACAACCAC | NM_001708.2 |
| *RHO* | TTTGGAGGGCTTCTTTGCCA | CCTCGGGGATGTACCTGGAC | NM_000539.3 |
