# Supplementary figures and images for: Developing a simple method to enhance the generation of cone and rod photoreceptors in pluripotent stem cell‐derived retinal organoids
Source: Stem Cells. 2019 Oct 31;38(1):45–51. doi: 10.1002/stem.3082 (PMC7004057; doi:10.1002/stem.3082)

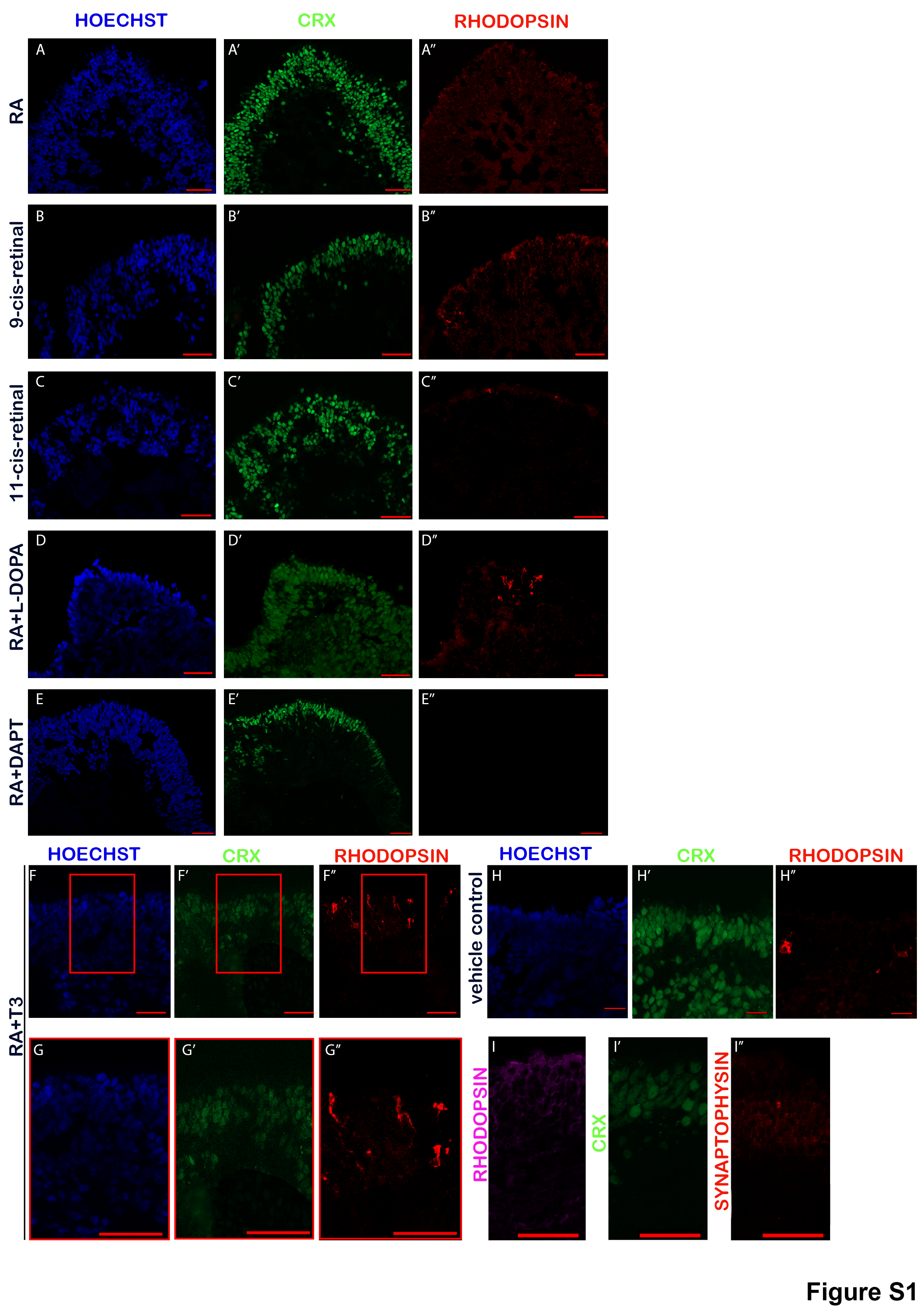

Supplement: Supplementary file 5 — Figure S1 Individual channels images of Rhodopsin immunoreactivity (red), CRX (green) represents the endogenous GFP expression and nuclei are counterstained with Hoechst (blue) in all conditions (A‐H) from day 90‐120 stage‐specific additions, showing the highest number of Rhodopsin+ cells in RA + T3 condition (F, F′ and F″). Higher magnification showed the nuclei in blue (G), the endogenous GFP expression in the apical layer (G’) and the typical morphology of rod photoreceptors (G”). Individual channel of the double staining with Rhodopsin (magenta) and Synaptophysin (red) indicated the possible formation of synapses in the developing OPL (I and I″); CRX (green) represents the endogenous GFP expression (I′). Scale bars 50 μm (A‐I″). [file STEM-38-45-s005.tif]

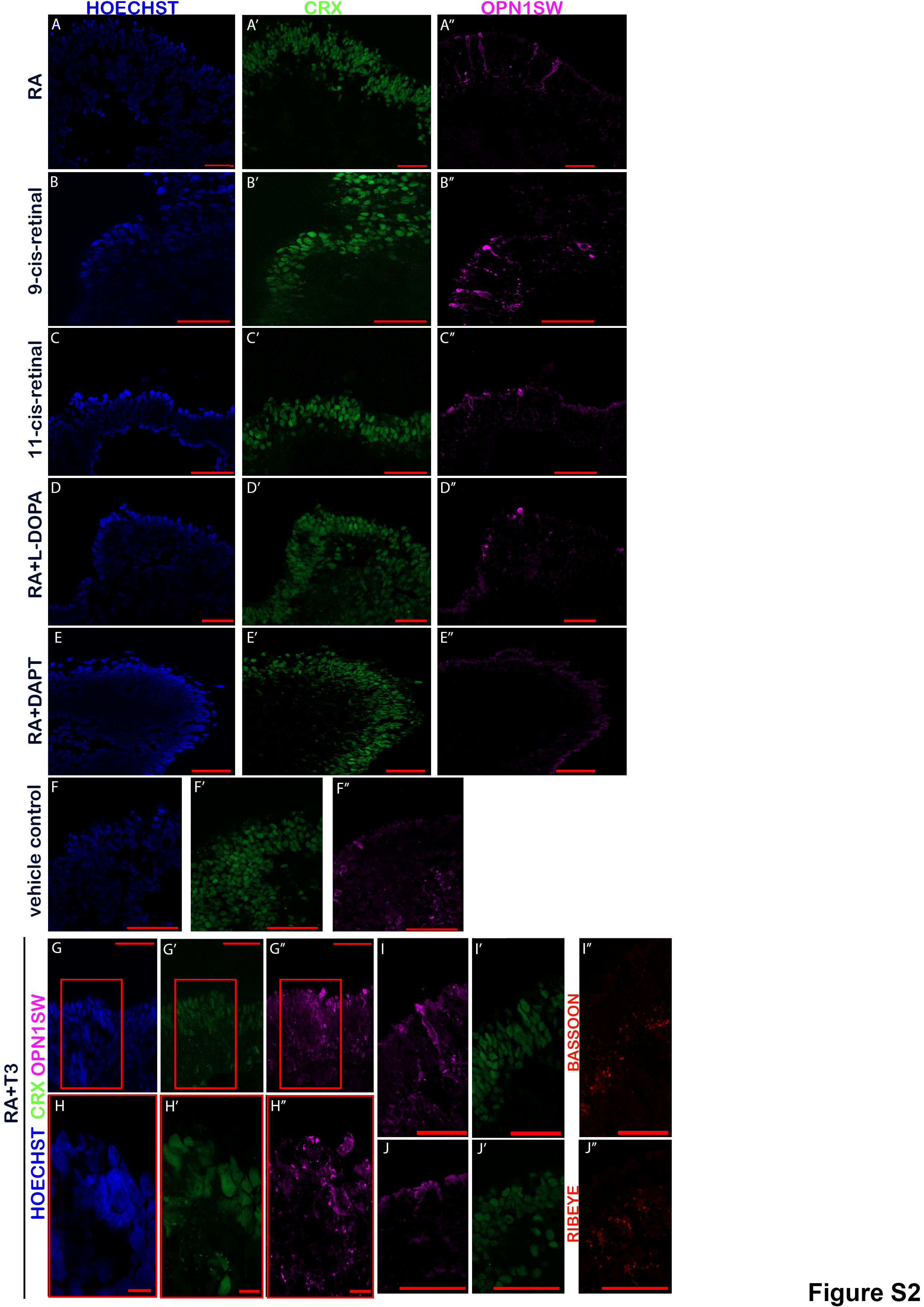

Supplement: Supplementary file 6 — Figure S2 Individual channels images of OPN1SW immunoreactivity (magenta), CRX (green) represents the endogenous GFP expression and nuclei are counterstained with Hoechst (blue) in all conditions (A‐H) from day 90‐120 stage‐specific additions, showing the highest number of OPN1SW+ cells in RA + T3 condition (G, G’ and G”). Higher magnification showed the nuclei in blue (H), the endogenous GFP expression in the apical layer (H′) and the typical morphology of S‐cone photoreceptors (H″). Individual channels of the double staining with OPN1SW (magenta) and Bassoon (red) (I and I″); OPN1SW (magenta) and Ribeye (red) (J and J”) indicated, respectively the possible formation of synapses in the developing OPL; CRX (green) represents the endogenous GFP expression (I′ and J’). Scale bars 50 μm (A, A’, A”, B, B′, B″, C, C′, C″, D, D’, D”, E, E’, E”, F, F′, F″, G, G’, G”, I, I′, I″, J, J’ and J”) and 10 μm (H, H′ and H″). [file STEM-38-45-s006.tif]

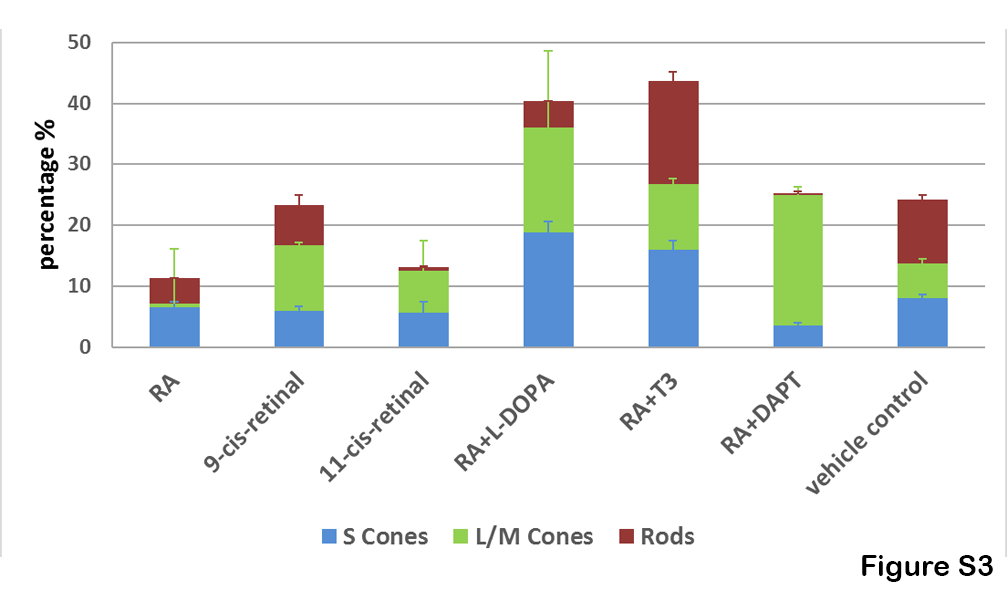

Supplement: Supplementary file 7 — Figure S3 Diagram showing the percentage of photoreceptors within the retinal organoids divided into S cones (blue), L/M cones (green) and rods (red) in all conditions. Data are presented as average of two time points: day 90‐120 and day 30‐120. [file STEM-38-45-s007.tif]

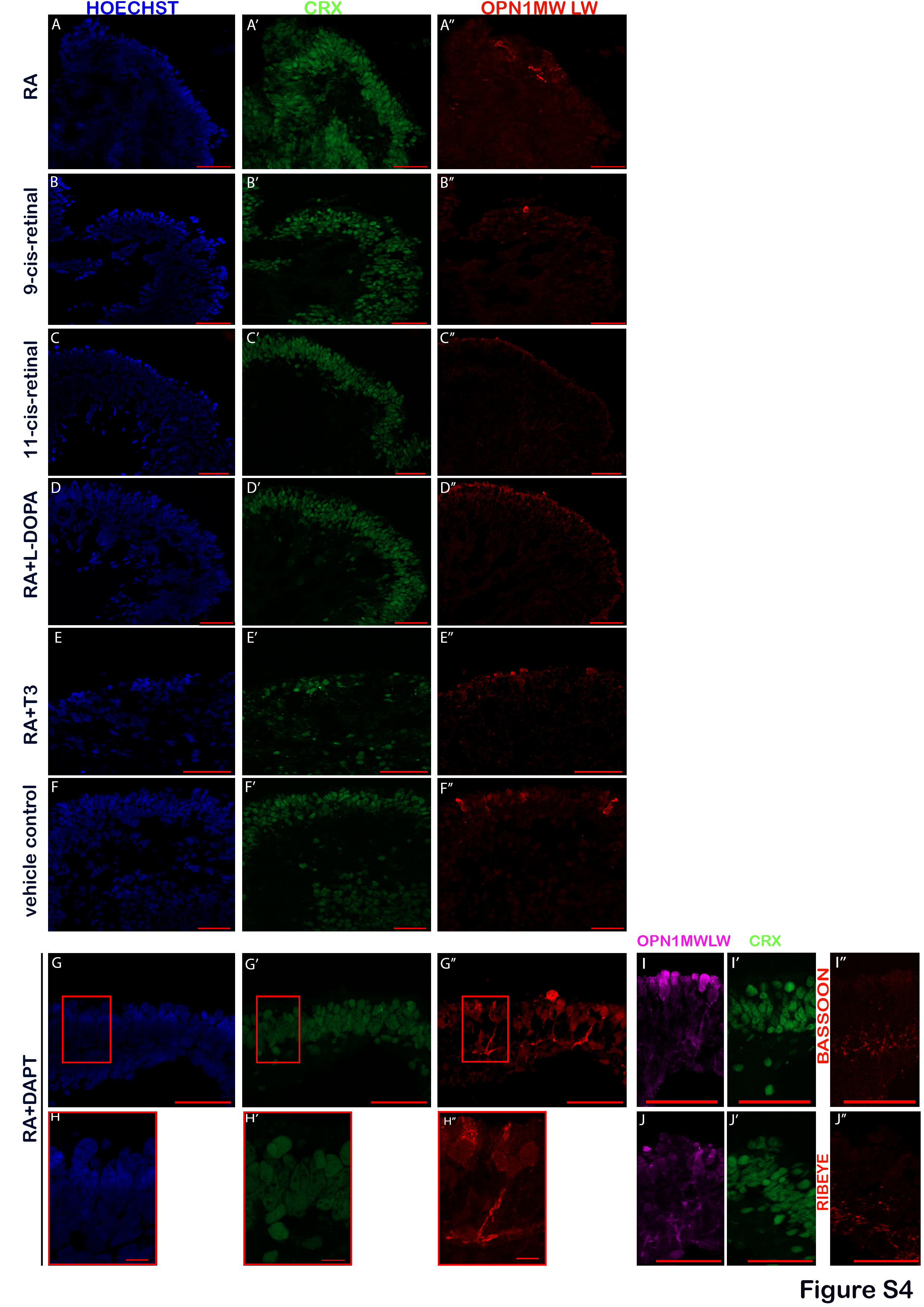

Supplement: Supplementary file 8 — Figure S4 Individual channels images of OPN1MW/LW immunoreactivity (red), CRX (green) represents the endogenous GFP expression and nuclei are counterstained with Hoechst (blue) in all conditions (A‐H) from day 30‐120 stage‐specific additions, showing the highest number of OPN1MW/LW+ cells in RA + DAPT condition (G, G’ and G”). Higher magnification showed the nuclei in blue (H), the endogenous GFP expression in the apical layer (H′) and the typical morphology of L/M‐cone photoreceptors (H″). Individual channels of the double staining with OPN1MW/LW (magenta) and Bassoon (red) (I and I″); OPN1MW/LW (magenta) and Ribeye (red) (J and J”) indicated the possible formation of synapses in the developing OPL. CRX (green) represents the endogenous GFP expression (I′ and J’). Scale bars 50 μm (A, A’, A”, B, B′, B″, C, C′, C″, D, D’, D”, E, E’, E”, F, F′, F″, G, G’, G”, I, I′, I″, J, J’ and J”) and 10 μm (H, H′ and H″). [file STEM-38-45-s008.tif]

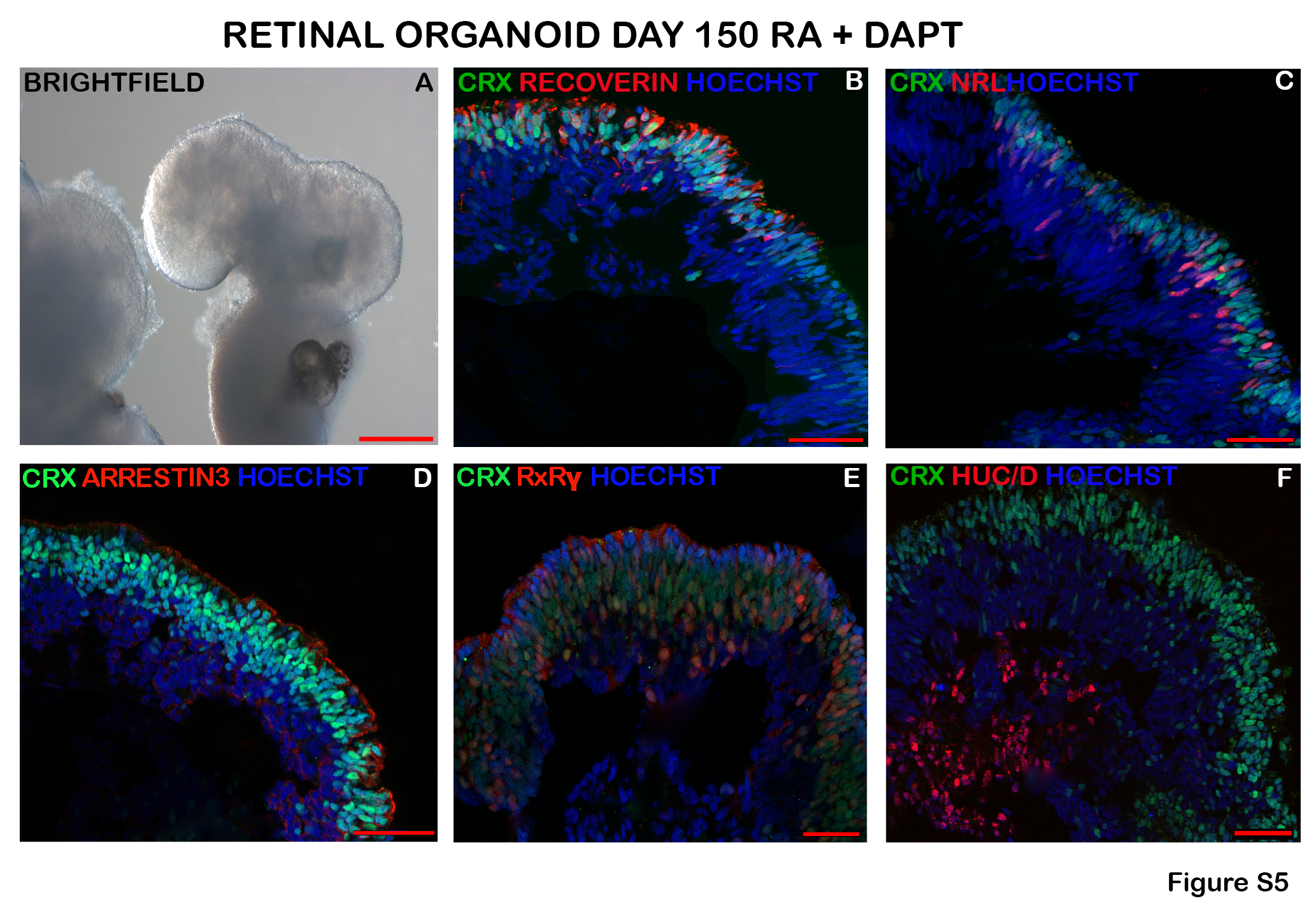

Supplement: Supplementary file 9 — Figure S5 Characterization of retinal organoid lamination in RA + DAPT condition at day 150 of differentiation. A) Representative brightfield images of retinal organoids at day 150, showing bright phase neuroepithelium on the apical side of organoids. B) CRX (endogenous GFP expression; green) and Recoverin (red) expression was found at the apical edge of organoids, forming a putative ONL. C) Some NRL+ cells (red) were found in the photoreceptor layer at the apical edge of retinal organoids. D) Expression of Arrestin3 (red) was observed above photoreceptor nuclei in the developing photoreceptor inner/outer segments. E) RXRγ+ cells (red) were seen throughout the retinal organoid. F) Ganglion cells detected by HuC/D (red) were located in the middle of retinal organoids, forming a putative GCL. CRX (green) represents the endogenous GFP expression and nuclei are counterstained with Hoechst (blue). Abbreviations: ONL, outer nuclear layer; GCL, ganglion cell layer. Scale bars, 200 pixel (A) and 50 μm (B‐F). Abbreviations: RA, Retinoic Acid. [file STEM-38-45-s009.tif]

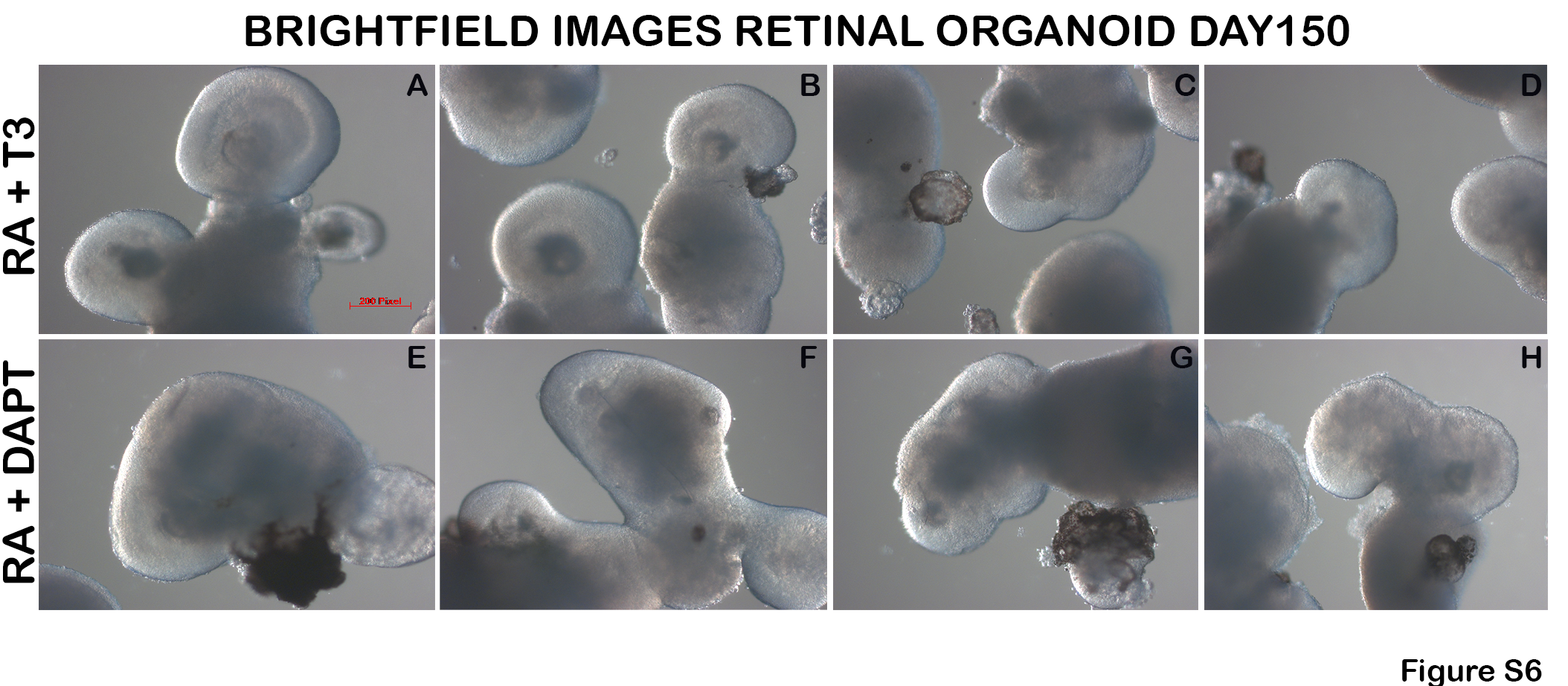

Supplement: Supplementary file 10 — Figure S6 Brightfield representative examples of retinal organoids at day 150 of differentiation for RA + T3 (A‐D) and RA + DAPT (E‐H) conditions. In both conditions, neural retinal and RPE are visible. Scale bar, 200 pixel. Abbreviations: RA, Retinoic Acid; T3, triiodothyronine. [file STEM-38-45-s010.tif]
